# Iron deficiency diagnosed using hepcidin on critical care discharge is an independent risk factor for death and poor quality of life at one year: an observational prospective study on 1161 patients

**DOI:** 10.1186/s13054-018-2253-0

**Published:** 2018-11-21

**Authors:** Sigismond Lasocki, Thibaud Lefebvre, Claire Mayeur, Hervé Puy, Alexandre Mebazaa, Etienne Gayat, on behalf of the FROG-ICU study group, N. Deye, C. Fauvaux, A. Mebazaa, C. Damoisel, D. Payen, E. Azoulay, A. S. Moreau, L. Jacob, O. Marie, M. Wolf, R. Sonneville, R. Bronchard, I. Rennuit, C. Paugam, J. P. Mira, A. Cariou, A. Tesnieres, N. Dufour, N. Anguel, L. Guerin, J. Duranteau, C. Ract, M. Leone, B. Pastene, T. Sharshar, A. Fayssoyl, J.-L. Baudel, B. Guidet, Q. Lu, W. Jie Gu, N. Brechot, A. Combes, S. Jaber, A. Pradel, Y. Coisel, M. Conseil, A. Veillard Baron, L. Bodson, Jy Lefrant, L. Elotmani, A. Ayral, S. Lloret, S. Pily-Flouri, Jb Pretalli, Pf Laterre, V. Montiel, Mf Dujardin, C. Berghe

**Affiliations:** 1 Département Anesthésie Réanimation, UBL Université, CHU Angers, 4 rue Larrey, 49933 Angers Cedex 9, France; 2 INSERM, UMR 1149/ERL CNRS 8252, Centre de Recherches sur l’inflammation, Université Paris Diderot, Paris, France; 3 Laboratoire d’excellence du Globule Rouge GR-Ex ; APHP, Hôpital Universitaire Louis Mourier, Colombes, France; 4 Department of Anesthesia, Université Paris Diderot ; U 942 Inserm ; APHP, Burn and Critical care, Hôpitaux Universitaires Saint louis – Lariboisiere, Paris, France

**Keywords:** Iron deficiency, Outcome, Critically ill, Quality of life, Hepcidin

## Abstract

**Background:**

Iron deficiency is difficult to diagnose in critically ill patients, but may be frequent and may impair recovery. Measurement of hepcidin could help in the diagnosis of iron deficiency. We aim to assess if iron deficiency diagnosed using hepcidin is associated with poorer outcome one year after an intensive care unit stay.

**Methods:**

We used the prospective FROG-ICU, multicentre (*n* = 28 ICUs), observational cohort study of critically ill survivors followed up one year after intensive care unit discharge. Iron deficiency was defined as hepcidin < 20 ng/l, ferritin < 100 ng/l or soluble transferrin receptor (sTfR)/log(ferritin) > 0.8, measured in blood drawn at intensive care unit discharge. Main outcomes were one-year all-cause mortality and poor quality of life (defined as a Short Form 36 (SF-36) score below the median).

**Results:**

Among the 2087 patients in the FROG-ICU cohort, 1570 were discharged alive and 1161 had a blood sample available at intensive care unit discharge and were included in the analysis. Using hepcidin, 429 (37%) patients had iron deficiency, compared to 72 (6%) using ferritin alone and 151 (13%) using the sTfR/log(ferritin) ratio. Iron deficiency diagnosed according to low hepcidin was an independent predictor of one-year mortality (OR 1.51 (1.10–2.08)) as was high sTfR/log ferritin ratio (OR = 1.95 (1.27–3.00)), but low ferritin was not. Severe ID, defined as hepcidin < 10 ng/l, was also an independent predictor of poor one-year physical recovery (1.58 (1.01–2.49)).

**Conclusions:**

Iron deficiency, diagnosed using hepcidin, is very frequent at intensive care unit discharge and is associated with increased one-year mortality and poorer physical recovery. Whether iron treatment may improve these outcomes remains to be investigated.

**Electronic supplementary material:**

The online version of this article (10.1186/s13054-018-2253-0) contains supplementary material, which is available to authorized users.

## Background

Iron deficiency (ID) is one of the main causes of chronic disease. ID affected more than 10% of the world’s population in 2015 and is responsible for almost half of all anaemia worldwide, which is the first cause of health impairment [1]. ID anaemia is indeed one of the leading causes of years of living with disability [1, 2]. However, ID is not only responsible for anaemia, it also causes fatigue and muscle weakness, independently of anaemia [3]. This has been notably described in patients with heart failure, for whom ID is associated with impaired cardiac function and increased mortality [4, 5]. ID may also be important in the prognosis of critically ill patients. First ID is frequent on admission in intensive care units (ICU), affecting 20–40% of the patients [6–8]. ID on admission has been associated with longer ICU stay, more renal failure or ICU-acquired infections [6, 8]. Because critically ill patients are exposed to frequent blood sampling and other causes of blood loss (including surgery, extracorporeal circuit, invasive procedures, etc.) [9, 10], they are exposed to high iron losses. Daily blood loss in these patients can be as high as 128 ml of blood per day [10], which represents 2–3 times the daily need for erythropoiesis [11]. ID is thus expected to be frequent on ICU discharge and may impair the post-ICU rehabilitation. Unfortunately, ID is difficult to diagnose using conventional markers (i.e. ferritin) in the presence of inflammation. Prevalence of ID on discharge is thus probably underestimated, with observed prevalence of less than 10% [12]. Many markers have been proposed to diagnose ID in the presence of inflammation [6–8, 13]. However, there is no consensus and the best markers (i.e. the percentage of hypochromic red cells) [14] are not usable in case of blood transfusion, which is very frequent in critically ill patients. Thus, the remaining proposed marker in this context is the ratio between soluble transferrin receptor and the log (ferritin) (sTfR/log ferritin) [15].

In recent decades, the understanding of iron metabolism has been greatly improved by the discovery of its master regulator, hepcidin [16]. A low hepcidin level may indicate ID in the critically ill patient [11, 17]. Because hepcidin may indicate ID and because ID is responsible for muscular weakness and fatigue, we hypothesised that ID diagnosed according to low plasma hepcidin or high sTfR/log(ferritin) on ICU discharge could predict a poorer outcome and quality of life (QOL) in critically ill patients. We used the prospective Frog-ICU cohort [18, 19] to determine whether ID diagnosis using these markers, measured at ICU discharge, were predictive of one-year mortality and QOL in critically ill patients discharged alive from ICU.

## Methods

We used data from the prospective observational cohort of the French and euRopean Outcome reGistry in Intensive Care Units (FROG-ICU). Briefly, this prospective study has been designed to better understand long-term outcome after ICU discharge and risk factors for morbidity and mortality [19]. Plasma was collected on ICU discharge and patients were followed for one year, with quality of life (QOL) assessment using the Short Form 36 (SF-36) questionnaire. Patients from 28 ICUs (and 19 hospitals in France and Belgium) were included if they were ventilated for more than 24 h or if they received catecholamine for more than 24 h. Patients were followed up on ICU discharge and at 3, 6 and 12 months after discharge [18]. The Ethical Committees waived the need for written consent; all patients and/or next of kin were informed and oral consent was documented in the patients’ medical records by the investigator. We followed the “Strengthening of reporting in observational studies in epidemiology” (STROBE) statement to report the results.

### Hepcidin measurement

Using a previously validated and published mass-spectrometry protocol [20], hepcidin concentration was measured in the blood sample obtained at ICU discharge. Briefly, after plasma pre-treatment by solid phase extraction, hepcidin was quantified using ultra high-pressure chromatography coupled to triple quadripole (Acquity UPLC-Xevo TQMS, Waters Guyancourt, France). Using this method, normal ranges for hepcidin concentration are between 1 and 20 ng/l. However, < 20 ng/l hepcidin concentration indicates ID in critically ill patients, as it corresponds to the threshold value determined using ELISA [17].

### Iron profile

An iron profile was determined in all patients, by measuring serum ferritin and soluble transferrin receptor (sTfR) using a Vista analyzer and NLatex sTfR, BNPprospec (Siemens, France). Iron deficiency was defined according to the usual definition in the presence of inflammation, by either ferritin < 100 ng/l or sTfR/log(ferritin) ratio > 0.8 [14, 15].

### Statistical analysis

All results are expressed as median with interquartile range or count with percentage. Marginal associations were tested using the chi-squared test and Mann-Whitney test as appropriate. The primary endpoint was to assess whether low hepcidin (i.e. hepcidin < 20 ng/l, corresponding to ID in the critically ill) predicts poorer outcome, defined as ICU discharge. To account for possible cofounders, we used logistic regression and adjusted this analysis according to the principal factors associated with one-year mortality in the Frog-ICU cohort (see [18] for complete description of multivariate analysis): age, gender, diabetes mellitus, liver disease, surgical/medical, septic shock and haemoglobin (Hb) at discharge. We also evaluated the predictive values of the other biological markers of ID, using the standard definitions (i.e. ferritin < 100 ng/l and sTfR/log(ferritin) > 0.8). Odds ratio (OR) and confidence interval (CI) at 95% was calculated as a measure of association. The log linearity of the quantitative variables was evaluated using restricted cubic splines.

A secondary aim was to evaluate if low hepcidin (i.e. presence of iron deficiency) predicts one-year QOL of these patients. For this purpose, we defined poorer QOL as a SF-36 score lower than the median observed SF-36 scores in our critically ill population, for the two dimensions (i.e. physical and mental). In order to complete our results, we conducted sensitivity analysis, using a lower threshold for ID (severe ID, defined as Hepcidin < 10 ng/l).

Because the diagnosis of ID may be modified in the presence of inflammation, we modelled the probability of one-year mortality according to the ID markers (i.e. hepcidin and sTfR/log(ferritin)) and the level of inflammation, by separating the population according to the different tertiles of interleukin-6 levels at discharge. A *p* value <0.05 was considered statistically significant. All statistical analyses were performed using R statistical software version 3.1.1 or above (The R Foundation for Statistical Computing, Vienna, Austria).

## Results

**Fig. 1:**
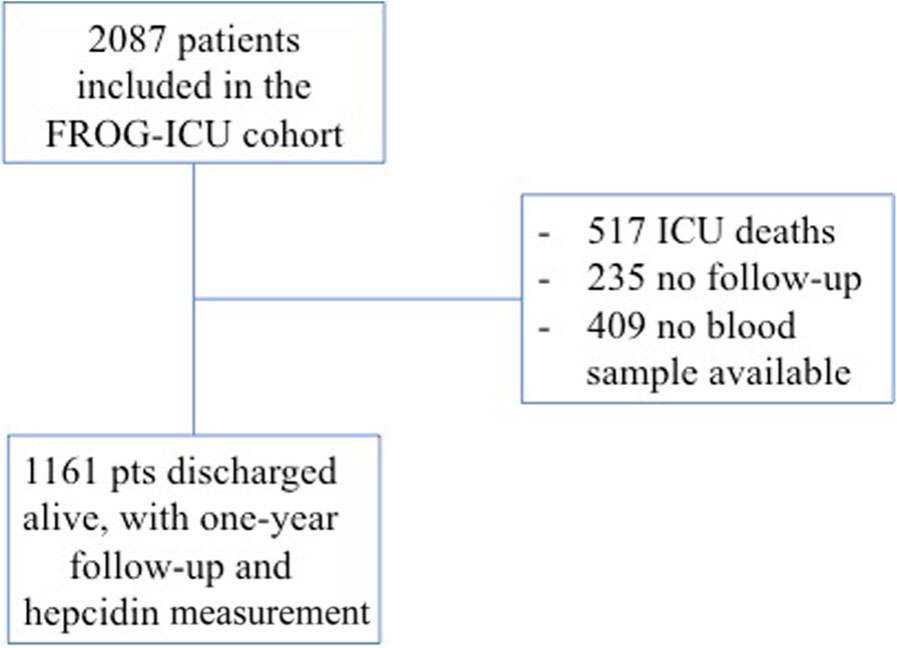
Study flow chart. ICU, intensive care unit; pts, patients

Between August 2011 and June 2013, 2087 patients were included in the FROG-ICU cohort, among them 1570 were discharged alive from the ICU and 1161 had a blood sample available together with one-year follow-up and were thus included in this study (Fig. 1).

### ID diagnosis

**Fig. 2:**
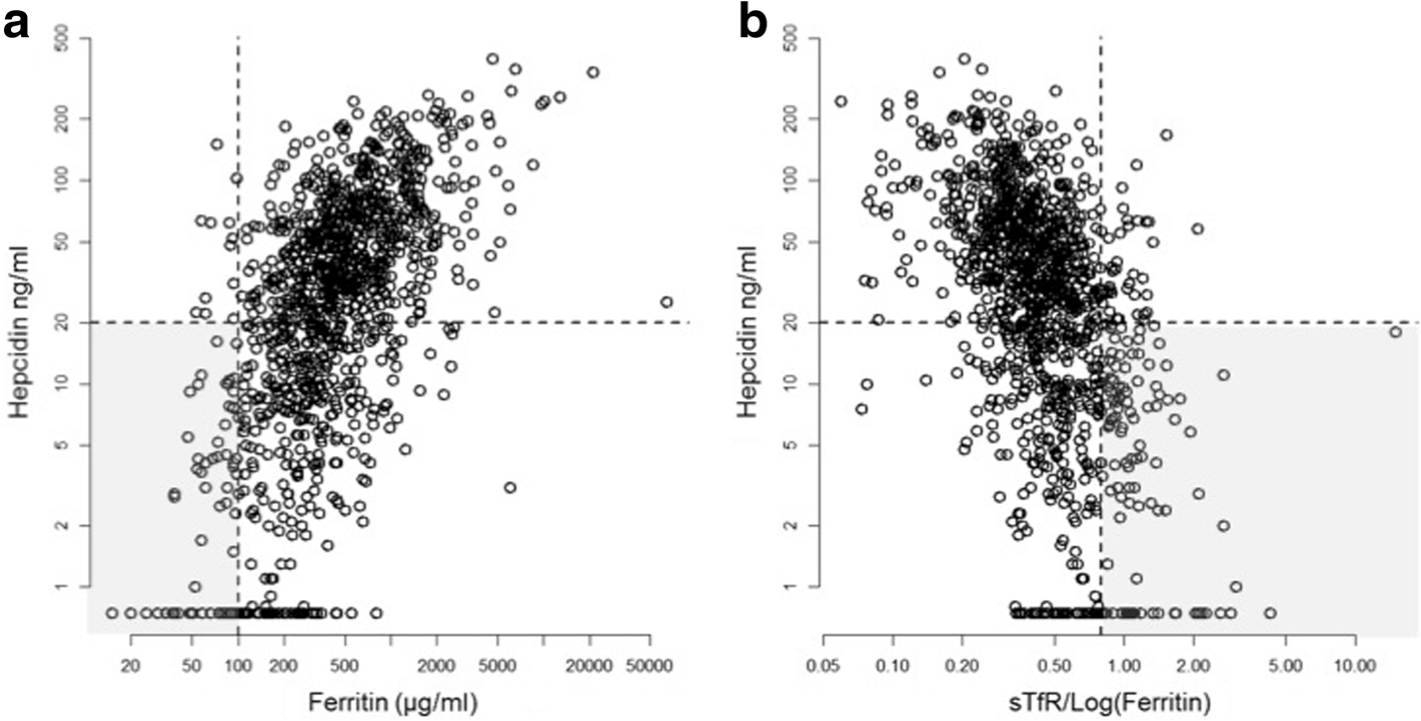
Relationship between hepcidin and ferritin (**a**) or soluble transferrin receptor (sTfR)/log(ferritin) (**b**). We identified positive correlation between log(hepcidin) and ferritin (**a**) (Pearson’s correlation coefficient [95% CI], 0.59 [0.55–0.62]) and negative correlation with sTfR/log(ferritin) (**b**). The concordance for iron deficiency (ID) diagnosis according to hepcidin and ferritin (**a**) was 67%, 95% CI 65–70% (i.e. the proportion of patients with hepcidin < 20 ng/l and ferritin < 100 ng/l or hepcidin > 20 ng/l and ferritin > 100 ng/l) and for sTfR/log(ferritin) (**b**) it was 71%, 95% CI 68–73%. Grey areas indicate the concordance between the different markers. Lower right (**a**) and left (**b**) panels indicate ID according to hepcidin that was not diagnosed according to ferritin (**a**) or sTfR/log(ferritin) (**b**). CRP, C-reactive protein

Hepcidin measurements were available for 1161 patients, ferritin for 1157 patients and sTfR/ferritin for 1146 patients. According to their hepcidin concentrations, 429 patients (37%) had ID (i.e. hepcidin < 20 ng/l) at ICU discharge. Based on ferritin, only 72 (6%) patients could be considered as having ID (i.e. ferritin < 100 ng/l). Based on the sTfR/log(ferritin) ratio, 151 (13%) patients had ID (i.e. sTfR/log(ferritin) > 0.8). Interestingly the concordance on ID diagnosis between hepcidin and ferritin was not so low (67%, 95% CI 65–70%) but was better with sTfR/(log ferritin) (71%, 95% CI 68–73%, Fig. 2). However, hepcidin identified more patients with ID (Fig. 2).

In our study population, hepcidin values were not correlated to Hb values, but there was positive correlation with CRP (and IL-6) and ferritin, and negative correlation with sTfR (Additional file 1: Figure S1). Patients with ID identified by hepcidin on (i.e. hepcidin < 20 ng/l) were more often women, had more liver disease and lower Hb at discharge, but their renal function was better than patients without ID. Patients with ID at ICU discharge had received fewer transfusion during their ICU stay (Table 1).

**Table 1:** Patients characteristics according to the diagnosis of ID (i.e. hepcidin < 20 ng/l)

	Non-ID (*n* = 732) (hepcidin ≥ 20 ng/l)	ID (*n* = 429) (hepcidin < 20 ng/l)	*p*
Age (years)	61 (50 to 72)	59 (47 to 72)	0.25
Women	240 (32.8)	183 (42.7)	0.00081
Charlson’s score	3 (1 to 4)	3 (1 to 4)	0.32
Diabetes mellitus	112 (15.3)	89 (20.7)	0.02
Chronic heart failure	41 (5.6)	34 (7.9)	0.14
Hypertension	268 (36.6)	185 (43.1)	0.029
Liver disease	31 (4.2)	44 (10.3)	< 0.001
Cancer	98 (13.4)	43 (10)	0.094
ICU admission			
SAPS II	46 (34.5 to 59)	46 (34 to 59)	0.87
SOFA	6 (4 to 9)	6 (4 to 9)	0.71
Septic shock	172 (23.5)	78 (18.2)	0.038
eGFR (ml/min)	82 (40.4 to 106)	87.9 (56.2 to 109)	0.0044
Hb (g/dl)	10.2 (9 to 11.6)	9.9 (9.1 to 11.1)	0.032
ICU stay			
ICU LOS (days)	13 (7 to 22)	12 (7 to 20)	0.19
Renal support *n* (%)	140 (19.1)	64 (14.9)	0.079
Catecholamine *n* (%)	547 (74.7)	294 (68.5)	0.025
Transfusion *n* (%)	343 (46.9)	162 (37.8)	0.0027
ICU discharge			
Hb (g/dl)	10.1 (9 to 11.3)	9.8 (8.9 to 10.8)	0.011
CRP (mg/l)	69.4 (32.2 to 117.3)	33 (13.9 to 66.7)	< 0.001
Ferritin (μg/l)	543.5 (339.8 to 932.2)	240 (138 to 366)	< 0.001
sTfR (mg/l)	1 (0.8 to 1.4)	1.4 (1 to 1.9)	< 0.001
sTfR/log ferritin	0.37 (0.28 to 0.51)	0.57 (0.43 to 0.85)	< 0.001
IL-6 (ng/l)	37.1 (19.6 to 70)	19.8 (9.4 to 38.4)	< 0.001
Outcome at one year			
- global score	56.9 (35.9 to 76.6)	53.6 (34.3 to 73.7)	0.21
- Mental QOL (MCS)	58.8 (36.9 to 77.9)	57.9 (35.9 to 77)	0.44
- Physical QOL (PCS)	54.7 (33.6 to 78.8)	50 (31.2 to 72.7)	0.11
One-year mortality	123 (16.8)	99 (23.1)	0.011

Data are expressed as median (Q1–Q3) or number (percentage)

*ID* iron deficiency, *ICU* intensive care unit, *SAPS II* Simplified Acute Physiology Score, *SOFA* sequential organ failure assessment, *eGFR* estimated glomerular filtration rate, *Hb* haemoglobin, *LOS* length of stay, *CRP* C-reactive protein, *sTfR* soluble transferrin receptor, *IL-6* interleukin 6, *QOL* quality of life

### ID and outcome

ID diagnosed by hepcidin was associated with one-year mortality (Table 1). ID diagnosed by sTfR/log(ferritin) ratio > 0.8, was also associated with higher mortality (17.7% vs 27.8%, *p* = 0.003), but ID defined by low ferritin (i.e. < 100 ng/l) was not (18.1% compared to 19.2% mortality, respectively, in patients with “low” and “high” ferritin, *p* = 0.93).

**Fig. 3:**
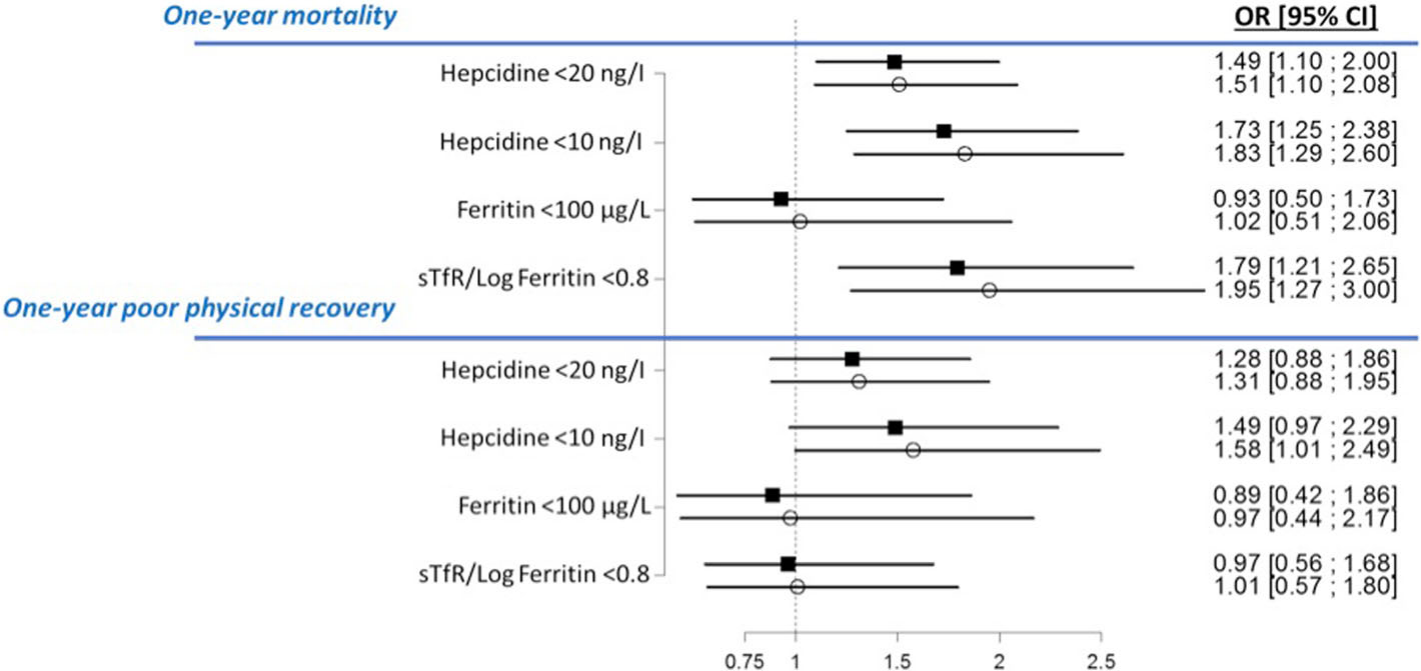
Odd ratio for one-year mortality and poor physical quality of life according to the different markers of iron deficiency. Squares indicate crude odd ratios and open circles indicate adjusted OR for main cofounding factors (age, gender, diabetes mellitus, liver disease, surgical/medical admission, septic shock and haemoglobin at discharge ). Poor physical recovery was defined as a physical component of the Short Form 36 score below the median value (i.e. 53) at one year. The SF-36 was available in 466 patients at one year. OR, odds ratio; 95% CI, 95% confidence interval, sTfR, soluble transferrin receptor

**Fig. 4:**
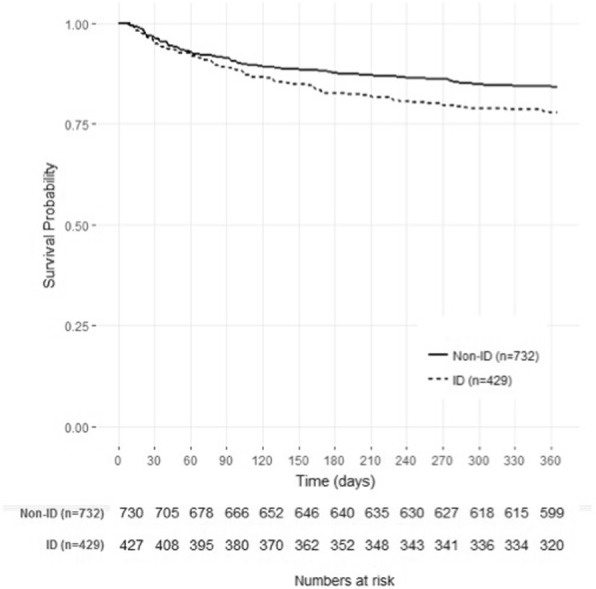
One-year survival distribution. Survival curves in patients with iron deficiency (ID) (dark line) and patients without ID (non-ID) (dotted line), according to hepcidin < or ≥ 20 ng/l at discharge) are shown from ICU discharge to day 360. Survival was longer in patients without ID (hazard ratio = 1.41 [1.10–1.89], *p* = 0.01)

The multivariate analysis adjusted for the main confounding factors confirmed that ID diagnosed by hepcidin is an independent predictor of one-year mortality as well as high sTfR/log ferritin ratio (Table 2 and Fig. 3). All the odds ratios from the multivariate analysis for the different definitions of ID are reported in Additional file 2: Table S1. Figure 4 shows the Kaplan-Meier survival curves of patients with and without ID, showing higher mortality in those with ID (hazard ratio (HR) = 1.41 (1.10–1.89), *p* = 0.01). These associations persisted when patients were separated according to their levels of inflammation (according to IL-6) (Additional file 3: Figure S2). The shape of the observed curves could indicate that a different threshold may be appropriate in the presence of inflammation (i.e. around 40–50 ng/ml for hepdicin) or in low inflammation (i.e. > 0.6 for the sTfR/log(ferritin) ratio (see Additional file 3: Figure S2)). In addition, severe ID defined as hepcidin < 10 ng/l was also independently associated with a poor physical QOL at one year, while the other ID markers were not (Fig. 3).

**Table 2:** Result of the multivariate model for the prediction of one-year post-ICU mortality

	OR [95% CI]	*P* value
Female gender	0.78 [0.56; 1.09]	0.14
Septic shock	1.27 [0.89; 1.81]	0.18
Diabetes mellitus	1.50 [1.04; 2.16]	0.03
Age (per year)	1.05 [1.04; 1.06]	< 0.001
ID (hepcidin < 20 ng/l)	1.51 [1.10; 2.08]	0.01
Chronic liver disease	2.51 [1.45; 4.33]	< 0.001

All the variables retained in the final model of the princeps publication were added to this model [18]

*OR* odds ratio, *CI* confidence interval, *ID* iron deficiency

## Discussion

In this prospective cohort of critically ill patients we observed that ID diagnosed by low hepcidin or high sTfR/log ferritin ratio is frequent at ICU discharge, affecting almost 40% of the patients and that ID is an independent predictor of one-year mortality. In addition, severe ID defined by hepcidin < 10 ng/l was also an independent predictor of one-year poor physical QOL.

This relatively high prevalence of ID is consistent with the prevalence observed in the general population (around 30–40% in menstruating women or preschool children) [21, 22] or in populations of patients such as those in the perioperative period (prevalence between 35 and 70%) [23] or patients with cardiac disease (prevalence around 40–50%) [24]. However, there are very few data available in critically ill patients, especially at ICU discharge. On admission to ICU, the prevalence of ID varies from 9% to almost 40% [6, 8, 17, 25–27]. These variations in ID prevalence are mainly related to the biological test that is used to diagnose ID. In the general population, ferritin is the gold standard for ID diagnosis [22]. However, ferritin synthesis is induced by inflammation, decreasing its interest as a diagnostic tool of ID in such conditions. Inflammation is present in virtually all critically ill patients and this probably explains why the prevalence of ID, diagnosed by low ferritin (i.e. ferritin < 100 μg/l) is < 10% on admission to ICU [27] or at discharge [12]. This is why other markers are proposed to diagnose ID in the presence of inflammation. Red blood cell indices, such as the percentage of hypochromic red cells or reticulocyte haemoglobin content are considered to be the best variables available to diagnose ID in the presence of inflammation [14]. Unfortunately, these parameters cannot be used to diagnose ID when patients receive blood transfusions, which is often the case in critically patients. sTfR and its ratio to log(ferritin) has been proposed to diagnose ID in the presence of inflammation [14, 15]. In this cohort, ID defined by a low sTfR/log(ferritin) ratio makes it possible to diagnose ID in an important number of critically ill patients discharged from ICU and these patients had a poor outcome. However, this assay has some limitations, it is quite expensive and is not standardized [14]. The reproducibility of measures remains uncertain. Hepcidin quantification appears promising and a number of laboratories are collaborating towards global harmonization of hepcidin assays [28]. The prevalence of ID identified by hepcidin that we observed in this cohort is consistent with the expected; as the prevalence of ID on admission is around 30–40%, the prevalence of ID on discharge is expected to be the same or higher due to frequent blood loss (even if ID on admission may be also associated with higher mortality). Because hepcidin is higher in patients who have received blood transfusions [29] and because around 40% of the patients have been transfused, it is possible that more patients had ID. In addition, hepcidin synthesis is linked to the iron stores and to the level of inflammation [11, 29, 30]. Hepcidin could also be useful to indicate when iron treatment could be necessary, in fact elevated hepcidin prevents the mobilization of iron from stores, by blocking the ferroportin, whereas low hepcidin allows the expression of ferroportin at the cell membrane of macrophages and thus the export of iron from stores [11, 30].

In this cohort, we observed that severe ID is associated with both increased one-year mortality and poorer physical QOL. This is also consistent with observations that ID is a risk factor for mortality and morbidity in patients with cardiac disease [4]. Indeed, ID has been shown to be responsible for decreased mitochondrial complex I activity, decreased exercise capacity and left ventricular ejection fraction in an animal model [31]. ID has been also associated with decreased muscular function (skeletal and myocardial) and this may explain the usual association reported between ID and fatigue [3, 12, 32].

Our study has some limitations. We used different definitions of ID, but we could also have used other markers such as erythrocyte zinc protoporphyrin [7, 25]. However, this marker may be influenced by blood transfusion and may not be suitable in these patients (at least at the end of their stay, with a transfusion rate around 40%). We observed an association between low hepcidin and liver disease, but we do not have enough data to further investigate this association. Although the association between ID and poor outcomes is supported by a physiological rationale, our data do not demonstrate that iron treatment is beneficial in these critically ill patients. Furthermore, we do not have blood samples after ICU discharge to determine whether some patients did recover from ID or not and if ID at 3, 6 or 12 months is still associated with poor outcomes. We also do not know if some of these patients have been treated with iron after ICU discharge, but this is not likely since iron is rarely proposed in these patients. We also could have investigated the link between high hepcidin and the outcomes. Indeed, we observed that high hepcidin was also associated with higher mortality (as shown in Additonal file 3: Figure S2 for the highest tertile), and some authors report that high hepcidin (on admission) is associated with blood transfusion for example [29]. But, although ID is treatable using iron, high hepcidin is not (because anti-hepcidin antibodies are not available yet). It is thus more indicative of more severe inflammation [29].

Interestingly, treatment of ID using intravenous iron has been proven to improve symptoms of fatigue, physical and mental QOL and even cardiac function [33, 34]. To date, iron treatment is not recommended for critically ill patients. However, we have already demonstrated in an animal model of critical care anemia with ID, that iron may be used to treat anemia, without toxicity (neither oxidative stress induction nor increased risk of infection) [35]. Some human data are promising. Intravenous iron does not induce more oxidative stress in critically ill patients compared to healthy volunteers [36], and it has been shown to improve haemoglobin level at hospital discharge [37]. The results of a prospective randomized controlled study (i.e. Hepcidan study, NCT02276690) [38] designed to assess whether diagnosing ID using this mass spectrometry hepcidin measurement on ICU discharge could improve the post-ICU rehabilitation of critically ill patients are expected soon and could help clinicians decide how to diagnose ID in the critically ill and whether iron treatment is useful.

## Conclusions

Iron deficiency, diagnosed using hepcidin concentration (< 20 ng/l) or sTfR/log(ferritin) ratio (> 0.8) at ICU discharge, is associated with increased one-year mortality. Severe ID (hepcidin < 10 ng/ml) is also an independent predictor of poor physical QOL at one year.

## Additional files

Additional file 1: **Figure S1.** Relationship between hepcidin and biological parameters. To better describe the relationship between hepcidin and biological parameters, we drew linear regressions between hepdicin (log transformed) and respectively haemoglobin (A), ferritin (B), C-reactive protein (CRP) (C), and soluble transferrin receptor (sTfR) (D). Pearson’s correlation coefficients [95% CI] were as follows: (A) 0.08 [0.02–0.14], (B) 0.59 [0.55–0.62], (C) 0.37 [0.32–0.42], (D) -0.37 [− 0.42 to −0.32]. (DOCX 389 kb)

Additional file 2: **Table S1.** Result of the multivariate model for the prediction of one-year post-ICU mortality according to the different ID definitions. All the variables retained in the final model of the princeps publication were added to this model [18]. OR, odds ratio; CI, confidence interval; ID, iron deficiency. (DOCX 19 kb)

Additional file 3: **Figure S2.** One-year mortality probability according to markers of iron deficiency and level of inflammation. Each panel indicates the relationship between hepcidin (a) and sTfR/log(ferritin) (b) at discharge and mortality probability according to the tertile of IL-6 at discharge. The blue arrows indicate the threshold values for iron deficiency diagnosis. This figure shows that the relationship between ID (defined as either low hepdidin or high sTfR/log(ferritin) ratio) persists with different level of inflammation. It also suggests that different threshold may be proposed in the presence of inflammation. sTfR, soluble transferrin receptor; IL-6, interleukin-6. (DOCX 55 kb)

## Abbreviations

CI: Confidence interval
ELISA: Enzyme-linked immunosorbent assay
Hb: Haemoglobin
ICU: Intensive care unit
ID: Iron deficiency
IL: Interleukin
OR: Odds ratio
QOL: Quality of life
SF-36: Short Form 36
sTfR: Soluble transferrin receptor
