# Supplementary material for: Iron deficiency diagnosed using hepcidin on critical care discharge is an independent risk factor for death and poor quality of life at one year: an observational prospective study on 1161 patients
Source: Crit Care. 2018 Nov 21;22:314. doi: 10.1186/s13054-018-2253-0 (PMC6249884; doi:10.1186/s13054-018-2253-0)

**Figure S1: Relationship between hepcidin and biological parameters**

To better describe the relation between hepcidin and biological parameters, we draw linear regressions between hepdicin (log transformed) and respectively (A) Haemoglobin, (B) Ferritin, (C) C-reactive protein (CRP), and (D) soluble transferrin receptor (sTfR).

Person’s correlation coefficient [95% CI] were as follows :

1. 0.08 [0.02 - 0.14]
2. 0.59 [0.55 - 0.62]
3. 0.37 [0.32 - 0.42]
4. -0.37 [-0.42 - -0.32]


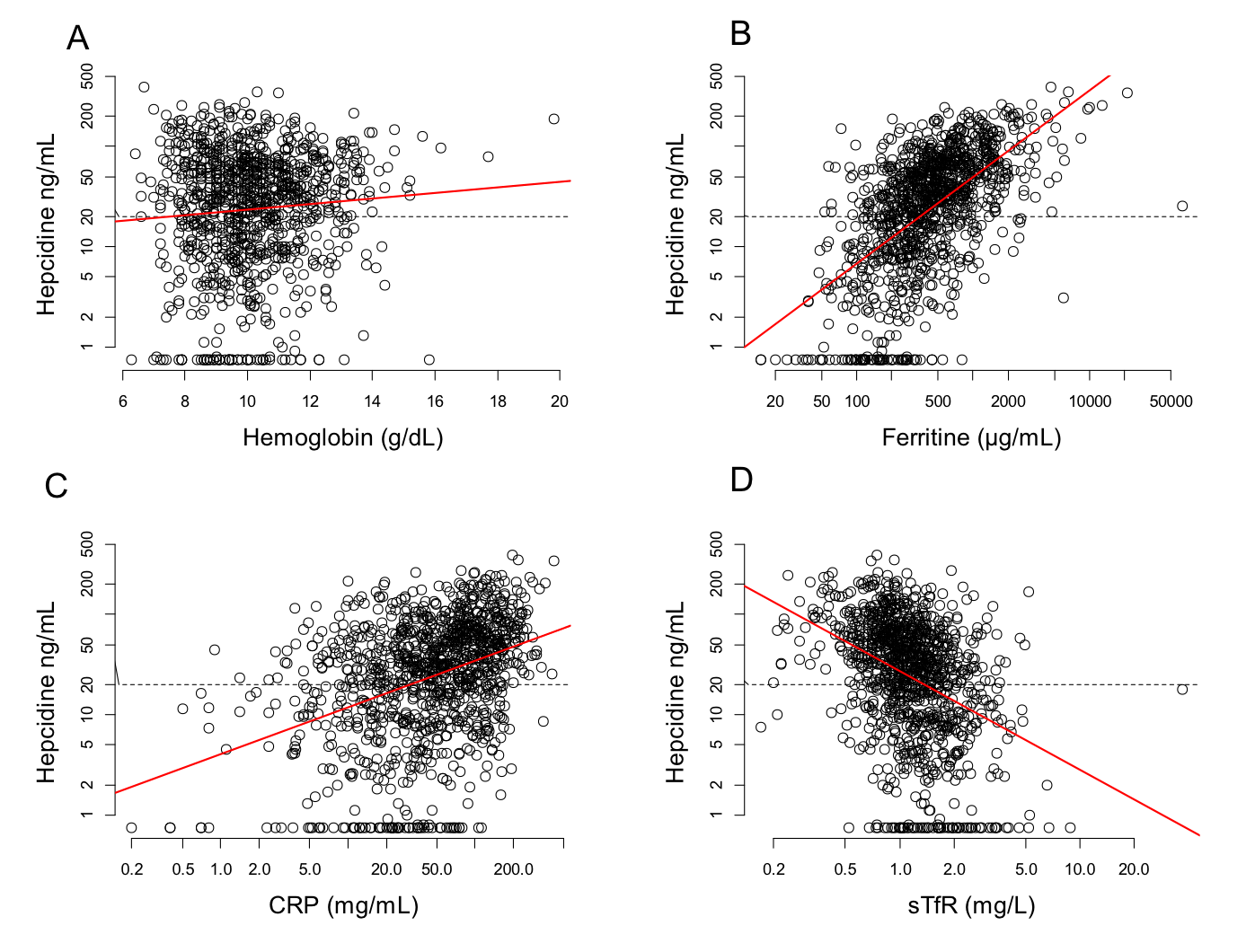

Supplement: Supplementary file 1 — Figure S1. Relationship between hepcidin and biological parameters. To better describe the relationship between hepcidin and biological parameters, we drew linear regressions between hepdicin (log transformed) and respectively haemoglobin (A), ferritin (B), C-reactive protein (CRP) (C), and soluble transferrin receptor (sTfR) (D). Pearson’s correlation coefficients [95% CI] were as follows: (A) 0.08 [0.02–0.14], (B) 0.59 [0.55–0.62], (C) 0.37 [0.32–0.42], (D) -0.37 [− 0.42 to −0.32]. (DOCX 389 kb) [file 13054_2018_2253_MOESM1_ESM.docx]
