# Supplementary material for: Iron deficiency diagnosed using hepcidin on critical care discharge is an independent risk factor for death and poor quality of life at one year: an observational prospective study on 1161 patients
Source: Crit Care. 2018 Nov 21;22:314. doi: 10.1186/s13054-018-2253-0 (PMC6249884; doi:10.1186/s13054-018-2253-0)
Supplement: Supplementary file 2 — Table S1. Result of the multivariate model for the prediction of one-year post-ICU mortality according to the different ID definitions. All the variables retained in the final model of the princeps publication were added to this model [18]. OR, odds ratio; CI, confidence interval; ID, iron deficiency. (DOCX 19 kb) [file 13054_2018_2253_MOESM2_ESM.docx]

**Result of the multivariate model for the prediction of one-year post-ICU mortality according to the different ID definitions**

All the variables retained in the final model of the princeps publication were added to this model [18].

OR, Odd ratio; CI, confident interval; ID, iron deficiency.

| ORs in the multivariable model | Definition of iron deficiency | | | |
| --- | --- | --- | --- | --- |
|  | **Hepcidin <20 ng/l** | **Hepcidin <10 ng/l** | **Ferritine <100 µg/l** | **sTfR/log(ferritin) >0.8** |
| **Iron deficiency** | 1.51 [1.10 ; 2.08] | 1.83 [1.29 ; 2.60] | 1.02 [0.51 ; 2.06] | 1.95 [1.27 ; 1.55] |
| **Age** | 1.05 [1.04 ; 1.06] | 1.05 [1.04 ; 1.06] | 1.05 [1.04 ; 1.06] | 1.05 [1.04 ; 1.01] |
| **Gender** | 0.78 [0.56 ; 1.09] | 0.78 [0.56 ; 1.09] | 0.81 [0.58 ; 1.12] | 0.80 [0.57 ; 1.60] |
| **Diabetes mellitus** | 1.50 [1.04 ; 2.16] | 1.43 [0.99 ; 2.07] | 1.57 [1.09 ; 2.26] | 1.50 [1.04 ; 1.49] |
| **Septic shock** | 1.27 [0.89 ; 1.81] | 1.29 [0.90 ; 1.83] | 1.20 [0.84 ; 1.71] | 1.31 [0.91 ; 1.72] |
| **Chronic liver disease** | 2.51 [1.45 ; 4.33] | 2.44 [1.42 ; 4.22] | 2.78 [1.62 ; 4.77] | 2.49 [1.44 ; 1.74] |
