# Supplementary material for: Iron deficiency diagnosed using hepcidin on critical care discharge is an independent risk factor for death and poor quality of life at one year: an observational prospective study on 1161 patients
Source: Crit Care. 2018 Nov 21;22:314. doi: 10.1186/s13054-018-2253-0 (PMC6249884; doi:10.1186/s13054-018-2253-0)

**Figure S2: One-year mortality probability according to markers of iron deficiency and level of inflammation**

Each panel indicates the relationship between discharge (a) hepcidin and (b) sTfR/log(ferritin) and mortality probability according the tertile of discharge IL-6. The blue arrows indicate the threshold values for iron deficiency diagnosis.

This figure shows that the relationship between ID (defined as either la ow hepdidin or a high sTfR/log(ferritin) ratio) persists for different level of inflammation. It also suggests that different threshold may be proposed in presence of inflammation.

sTfR, soluble transferrin receptor; IL-6, interleukin-6


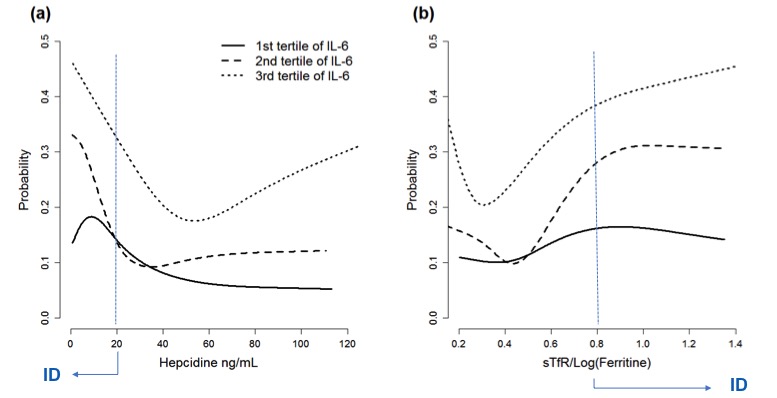

Supplement: Supplementary file 3 — Figure S2. One-year mortality probability according to markers of iron deficiency and level of inflammation. Each panel indicates the relationship between hepcidin (a) and sTfR/log(ferritin) (b) at discharge and mortality probability according to the tertile of IL-6 at discharge. The blue arrows indicate the threshold values for iron deficiency diagnosis. This figure shows that the relationship between ID (defined as either low hepdidin or high sTfR/log(ferritin) ratio) persists with different level of inflammation. It also suggests that different threshold may be proposed in the presence of inflammation. sTfR, soluble transferrin receptor; IL-6, interleukin-6. (DOCX 55 kb) [file 13054_2018_2253_MOESM3_ESM.docx]
